# Exploring the longitudinal relationship between physical activity and grit: the mediating role of creative personality in middle school students

**DOI:** 10.3389/fpubh.2026.1834779

**Published:** 2026-06-12

**Authors:** Inwoo Kim, Sujin Kim

**Affiliations:** Department of Sports Science Convergence, Dongguk University, Seoul, Republic of Korea

**Keywords:** adolescent development, creative personality, grit, latent growth modeling, physical activity

## Abstract

**Background:**

Grit is a critical determinant of long-term success during adolescence, yet the developmental pathways associated with developing this trait through physical activities remain underexplored.

**Objective:**

This study investigated the longitudinal relationships among exercise time, creative personality, and grit, specifically examining whether creative personality mediates the developmental link between physical activity and grit in middle school students.

**Methods:**

Data were drawn from the Korean Children and Youth Panel Survey (KCYPS 2018), including 2,325 middle school students tracked over three waves. A latent growth mediation model was employed to assess the longitudinal associations between the developmental trajectories of exercise time and grit, with creative personality as a mediating variable.

**Results:**

Exercise time was positively associated with both creative personality and grit at their baseline levels. Trajectories of exercise time were significantly associated with the patterns of creative personality and grit over the three-year period. Mediation analyses indicated that creative personality significantly mediated the relationship between exercise participation and grit development. Specifically, engagement in physical activity was associated with a more sustained creative personality, which in turn was associated with a favorable trajectory for grit among adolescents.

**Conclusion:**

Physical activity may serve as a supportive factor for supporting grit by being positively linked to creative personality during early adolescence. These findings suggest that to promote youth resilience and goal orientation, practitioners and educators could consider integrated programs that combine structured physical engagement with opportunities for fostering creative traits.

## 1 Introduction

In a world increasingly defined by challenges and uncertainties, the ability to persist in the face of adversity has never been more critical. This tenacity, encapsulated by the psychological construct of grit, has become a focal point in understanding the drivers of long-term success (1). Defined as the perseverance and passion for achieving sustained goals, grit offers a powerful lens through which to examine how individuals navigate obstacles and maintain focus amidst distractions (1, 2). It is this combination of unwavering effort and consistent interest that sets grit apart as a critical determinant of success.

For adolescents, the development of grit is particularly important, as it equips them with the resilience and determination needed to navigate the challenges of academic, social, and emotional growth (3). Studies have shown that grit is closely associated with academic achievement, emotional well-being, and the ability to manage setbacks effectively during this formative stage (4, 5). Beyond academics, grit is linked to essential life skills such as perseverance, self-regulation, and resilience, which enable adolescents to thrive in extracurricular pursuits and social relationships (6–8). By focusing on grit as a key developmental trait, educators and policymakers can better address the needs of adolescents, preparing them to excel in both academic and personal domains. Highlighting the importance of grit for middle school students emphasizes its critical role in shaping positive outcomes and underscores the need to identify effective strategies associated with this essential trait (7).

Despite its prominence, the conceptualization of grit has faced significant criticism regarding its distinctiveness from conscientiousness. Critics argue that if grit overlaps with existing personality traits, its incremental validity as a distinct construct remains questionable (6). These debates, however, have primarily been centered on cross-sectional findings. By employing a longitudinal framework (9), the current study seeks to address these concerns by examining the developmental trajectories of grit.

Physical activity serves as a dynamic and supportive factor for grit among adolescents, offering structured opportunities to build resilience, perseverance, and self-discipline (10). The structured nature of sports and exercise enables adolescents to face challenges, set goals, and persist through adversity, all of which align closely with the psychological traits that define grit (11, 12). Research has increasingly highlighted the connection between physical activity and grit development. Participation in physical activities inherently involves goal setting, accomplishment, and resilience-building experiences (13). For instance, sports provide opportunities to set measurable objectives, such as achieving a personal record or mastering a skill, which are associated with the kind of sustained effort characteristic of grit (14). Such experiences nurture resilience, a key component of grit, as demonstrated by studies emphasizing the protective role of physical activity in stress-related resilience (15).

While evidence supports the positive association between the physical activity and grit, the underlying pathways remain underexplored. Emerging research suggests the existence of mediating variables, such as self-esteem, social connectedness, and psychological resilience, which could illuminate how physical activity relates to grit. For example, studies indicate that creative personality, often supported through physical activities, may act as a mediating factor, linking athletic engagement to the development of grit. A study highlights that physical activity is positively associated with creative personality in children and youths, supporting its role in fostering cognitive and emotional development (16). Additionally, research demonstrates that chronic physical activity can significantly support creative personality over time, providing further evidence of its mediating role (17).

Creative personality has emerged as a significant psychological trait with profound implications for success during adolescence, particularly within academic and social domains (18). Defined as the dispositional ability to generate novel and useful ideas, creative personality encompasses divergent thinking processes, including originality, fluency, and flexibility (19). Creative personality is widely recognized as a reliable indicator of creative potential, with its components serving as predictors of creative achievements in educational and extra-curricular activities (19–21). These dispositional skills play a critical role in academic performance, problem-solving, and healthy psychological functioning during adolescence (22, 23).

Adolescence is a critical period for fostering creative personality, and engaging in physical activities offers an effective avenue for this development. Physical activity, particularly in the form of structured sports and exercises, provides rich environments that stimulate creative personality. Evidence suggests that participation in sports is linked to enhanced creative personality by exposing adolescents to dynamic and unpredictable environments that require problem-solving, strategic thinking, and adaptability (24). Team sports, for example, are associated with creative personality through the necessity to anticipate opponents’ strategies and coordinate effectively with teammates (25). Likewise, unstructured activities, such as recreational play, encourage imaginative exploration and spontaneous decision-making, both of which are critical for maintaining creative personality (26, 27).

Furthermore, the iterative nature of creative processes requires resilience in the face of failure, an attribute closely linked to grit. Research has shown that feedback in creative problem-solving activities is associated with persistence and adaptability, traits that are critical for overcoming challenges and aligning with the concept of grit (28). Ballerini et al. (29) showed that creative activities are linked to creative personality while supporting grit, highlighting the potential for such interventions in promoting resilience and sustained goal orientation. These programs suggest that supporting creative personality can simultaneously strengthen traits associated with grit.

The mediating role of creative personality in the relationship between physical activity and grit is both plausible and significant. Engaging in creative processes through sports and exercise provides individuals with opportunities to develop creative personality and the resilience needed to persist in achieving long-term goals. Evidence supports the notion that participation in activities requiring creative problem-solving is associated with intrinsic motivation, which serves as a key driver of both creative personality and grit (30, 31). While further empirical research is needed to elucidate this relationship, existing studies indicate that creative personality serves as a critical pathway for translating the benefits of physical activity into grit development. By framing creative personality as a mediating variable, this study bridges the gap between physical activity and grit, offering valuable insights into the interactions among non-cognitive skills and practical strategies for supporting resilience and goal orientation in youth.

To provide a cohesive explanation for these associations, the current study adopts the ‘Conceptual Model of Health through Sport’ (13) as its integrated theoretical framework, grounded in the principles of Positive Youth Development (PYD) (12, 14). This model posits that participation in physical activities facilitates a ‘transfer’ of structural experiences into psychological assets (13, 15). Specifically, the dynamic and unpredictable nature of sports requires constant creative personality and strategic problem-solving, which foster the core components of a creative personality (24). According to this framework, such developed psychological resources do not remain isolated but function as internal strengths that support broader non-cognitive skills. In this context, creative personality serves as a vital cognitive-emotional resource that enables adolescents to navigate obstacles and maintain long-term goals, thereby fostering the development of grit (29). By utilizing this integrated model, the present study moves beyond examining simple correlations and illuminates the sequential mechanism through which physical engagement builds the psychological foundation necessary for sustained perseverance during early adolescence.

While existing research has established links between physical activity, creative personality, and grit, most studies remain cross-sectional, limiting their ability to capture developmental changes over time. Cross-sectional studies fail to address the iterative processes through which physical activity may relate to creative personality and grit during adolescence. Longitudinal studies, however, can examine how these relationships evolve and provide insights into potential mechanisms. By tracking changes in exercise time, creative personality, and grit, longitudinal approaches can illuminate whether physical activity is associated with sustained improvements in creative personality and how these associations contribute to the development of grit (9, 32).

In addressing these research gaps, it is necessary to acknowledge the methodological limitation of using a single-item self-report measure for physical activity (weekly exercise time). While single-item indicators may not capture the full multidimensionality or intensity of exercise compared to comprehensive scales (33), they have been empirically validated for their reliability and practical utility in large-scale longitudinal studies (34). By utilizing this measure within a Latent Growth Mediation Model, this study provides a robust estimation of how general patterns of physical activity relate to the developmental trajectories of grit over a three-year period.

The current study seeks to address these gaps by employing a longitudinal mediation model to investigate the relationships among weekly physical activity, creative personality, and grit in middle school students. This approach allows for a nuanced exploration of how changes in exercise time relate to grit both directly and indirectly through creative personality. Furthermore, this study contributes to the broader understanding of non-cognitive skills during adolescence, offering valuable implications for educational programs and policies targeting holistic development in youth.

### 1.1 Research hypotheses

Guided by the ‘Conceptual Model of Health through Sport’ (13) and the cumulative gain perspective (14), the following hypotheses were formulated to test the specific longitudinal mechanisms between physical activity, creative personality, and grit. We theorize that initial exercise levels reflect immediate psychological resources, while sustained changes in exercise time function as a longitudinal trigger for cultivating adaptive cognitive mechanisms. Accordingly, the following hypotheses are proposed:

*H*1. Baseline levels of weekly exercise time will be positively associated with baseline levels of both creative personality and grit.

*H*2. The initial level and the rate of change in exercise time will be positively associated with the initial level and the rate of change in grit over the three-year period.

*H*3. Creative personality will significantly mediate the longitudinal relationship between exercise time and grit. Specifically, the developmental trajectory of exercise time will be associated with the maintenance or development of creative personality, which in turn will be positively linked to the developmental trajectory of grit.

## 2 Methods

### 2.1 Data source and participants

This study analyzed secondary data from the Korean Children and Youth Panel Survey 2018 (KCYPS 2018), a longitudinal research project managed by the National Youth Policy Institute (NYPI) in Korea. The KCYPS 2018 utilized a multi-stage stratified cluster sampling design to establish national representativeness, deliberately structured to capture generalized nationwide trends rather than school-specific effects. The sample was proportionally allocated across 17 provinces based on student populations, with strata further refined by regional scale (urban vs. rural) to minimize localized bias. Within each stratum, sample schools were selected using probability proportional to size (PPS) sampling, after which one class was randomly sampled from each school for full enumeration.

For the current study, data were drawn from the middle school cohort spanning the first three survey waves (2018–2020). To rigorously handle longitudinal missing data without sacrificing statistical power or inducing selection bias, multiple imputation (MI) was performed. This approach retained the entire baseline cohort, yielding a final robust sample of 2,590 students (1,405 boys and 1,185 girls).

To empirically validate the use of a single-level Latent Growth Modeling (LGM) framework, Intraclass Correlation Coefficients (ICCs) were calculated using a Linear Mixed Model across all primary variables. The calculated ICCs were exceptionally low, ranging from 0.025 to 0.125 (specifically, 0.079 to 0.125 for physical activity, 0.025 to 0.047 for creative personality, and 0.026 to 0.042 for grit). Since these values fall well below the conventional threshold of 0.10, between-school variances were deemed negligible. Thus, a single-level LGM approach was justified, ensuring both model parsimony and estimation stability.

### 2.2 Measurements

#### 2.2.1 Exercise time

Participants’ exercise time was assessed in the KCYPS 2018 using a single-item question adapted from the International Physical Activity Questionnaire (IPAQ) framework (33). This item specifically inquired about the amount of time spent engaging in moderate-intensity physical activities that induced sweating over the past week. Responses were recorded on a 5-point Likert scale, with options ranging from 1 (“None”) to 5 (“4 h or more”). This measure provided a straightforward method to capture weekly exercise duration for the longitudinal analysis. In the latent growth modeling framework, this 5-point measure was treated as a continuous variable, as the data demonstrated a relatively normal distribution suitable for structural equation modeling (35). This approach provided a straightforward and statistically viable method to capture weekly exercise duration for the longitudinal analysis.

#### 2.2.2 Creative personality

The assessment of creative personality utilized data from the KCYPS 2018, derived from a scale adapted by Choe and Pyo (36) based on the Creative Personality Scale (CPS) initially developed by Gough (37). The CPS includes 30 descriptive adjectives, of which 18 are indicative of creative characteristics and 12 denote non-creative traits. Participants were asked to choose the adjectives that they felt best described themselves. In this assessment, each selected creative trait was assigned a score of +1, while non-creative traits were scored as −1. The aggregate score ranged from −12 to 18, with higher scores reflecting a greater tendency toward creative personality traits. The total scores provided in the KCYPS 2018 dataset were directly used for the analysis.

#### 2.2.3 Grit

Grit was measured using the Korean Version of the Grit Scale for Children, developed and validated by Kim and Hwang (38) based on Duckworth’s original scale (2). The scale consists of two subdimensions: perseverance of effort (4 items) and consistency of interest (4 items), totaling 8 items. An example item for the consistency of interest dimension is, “I sometimes lose interest in a task shortly after starting it,” while an example item for the perseverance of effort dimension is, “I always finish what I start.” Responses were recorded on a 4-point Likert scale (1 = Not at all true, 4 = Very true). The internal consistency reliability (Cronbach’s alpha) for the grit scale was 0.712 at the first wave, 0.685 at the second wave, and 0.701 at the third wave, indicating acceptable reliability across all three time points.

### 2.3 Statistical analysis

This study conducted statistical analyses to explore the relationships among exercise time, creative personality, and grit, utilizing IBM SPSS Statistics 26 for descriptive and correlation analyses. To handle missing data rigorously, statistical analyses were performed on the dataset generated through Multiple Imputation (MI), preserving the full baseline sample of 2,590 students.

To examine the longitudinal mediation model, Latent Growth Modeling (LGM) was applied using the AMOS 21.0 software. LGM is a robust analytical method that enables simultaneous examination of associations among variables and their developmental trajectories over time, offering insights into both initial levels (intercepts) and rates of change (slopes) for the variables of interest (39).

The model was estimated using the Maximum Likelihood (ML) method. For the latent growth parameters, the factor loadings for the intercepts were fixed at 1.0, and the loadings for the slopes were fixed at 0, 1, and 2 to represent a linear change over the three-year period. The justification for the linear change model was based on the observed mean trends across the waves. Furthermore, as the KCYPS 2018 employs standardized and pre-validated measurement packages consistently across all waves, longitudinal measurement invariance was strictly sustained by design. This rigorous longitudinal framework ensures that the captured changes in latent growth parameters reflect actual developmental shifts in students rather than psychometric or structural artifacts.

The longitudinal mediation model was constructed to investigate the associations among the initial values of exercise time, creative personality, and grit, as well as their respective growth rates. This model enabled the identification of direct and indirect pathways through which exercise time was linked to grit, with creative personality as a potential mediator. To account for measurement dependency in the longitudinal data, residuals of the same indicators were allowed to correlate across time. Finally, to evaluate the significance of the mediating effects, the bootstrapping method was employed with 5,000 iterations to generate bias-corrected confidence intervals for indirect effects. This method ensured the robustness of the findings by providing a reliable estimation of mediation effects (40). The confidence interval was set at 95%, and the significance level (*α*) was established at 0.05.

## 3 Results

### 3.1 Descriptive statistics

The descriptive statistics for exercise time, creative personality, and grit across the three measurement points are presented in Table 1. The mean values of exercise time exhibited a consistent linear decline over time, decreasing from 3.032 at Wave 1 to 2.809 at Wave 2, and further down to 2.315 at Wave 3. Similarly, creative personality scores showed a gradual downward trend, with mean values of 1.446 at Wave 1, 1.097 at Wave 2, and 1.087 at Wave 3. In contrast, grit remained relatively stable across the three longitudinal time points, displaying mean values of 2.657 at Wave 1, 2.627 at Wave 2, and 2.570 at Wave 3. Crucially, because the observed mean trends for all primary constructs demonstrated consistent, monotonic linear adjustments across the three waves, the linear growth assumption was empirically justified, confirming that a linear parameterization was the most appropriate framework for the subsequent latent growth modeling.

**Table 1:** Descriptive statistics.

Variables	*M*	*SD*	Skewness	Kurtosis
Exercise time w1	3.032	1.388	0.057	−1.135
Exercise time w2	2.809	1.396	0.220	−1.183
Exercise time w3	2.315	1.373	0.589	−1.026
Creative personality w1	1.446	3.142	0.730	−0.638
Creative personality w2	1.097	2.887	0.778	−0.324
Creative personality w3	1.087	2.908	0.771	−0.354
Grit w1	2.657	0.438	0.160	−0.344
Grit w2	2.627	0.402	0.233	−0.117
Grit w3	2.570	0.418	0.147	−0.198

*N* = 2,325. w1 = wave 1, w2 = wave 2, w3 = wave 3.

To evaluate the normality of the data, skewness and kurtosis were assessed based on Kline’s thresholds (35), where acceptable skewness values are ≤ 3.0 and kurtosis values are ≤ 8.0. Empirically, the skewness values for all primary variables across the three waves ranged from 0.057 to 0.778, while the kurtosis values ranged from −1.363 to 0.963. These findings demonstrate that all indicators fall well within the permissible limits, confirming that the data successfully satisfy the normality assumptions required for subsequent maximum likelihood estimations in Latent Growth Modeling.

### 3.2 Correlation analysis

The correlation analysis revealed significant relationships among exercise time, creative personality, and grit across the three measurement periods (Table 2). Pearson correlation coefficients indicated that exercise time demonstrated moderate and significant stability over time, with coefficients ranging from *r* = 0.293 to *r* = 0.403 (*p* < 0.01). Creative personality also exhibited strong longitudinal stability across waves (*r* = 0.316 to *r* = 0.408, *p* < 0.01) and maintained positive associations with exercise time (*r* = 0.049, *p* < 0.05 to *r* = 0.178, *p* < 0.01). Furthermore, grit showed robust stability over the three-year period (*r* = 0.409 to *r* = 0.467, *p* < 0.01). Crucially, grit was consistently and positively correlated with both exercise time (*r* = 0.090 to *r* = 0.190, *p* < 0.01) and creative personality (*r* = 0.104 to *r* = 0.219, *p* < 0.01) across all measurement points. These significant bivariate interrelations across waves provide empirical justification for the structural path dependencies, confirming the theoretical viability of establishing the subsequent longitudinal mediation model.

**Table 2:** Correlation matrix.

Variables	1.	2.	3.	4.	5.	6.	7.	8.
Exercise time w1	—							
Exercise time w2	0.403∗∗	—						
Exercise time w3	0.293∗∗	0.330∗∗	—					
Creative personality w1	0.175∗∗	0.112∗∗	0.049∗	—				
Creative personality w2	0.172∗∗	0.178∗∗	0.123∗∗	0.408∗∗	—			
Creative personality w3	0.078∗∗	0.096∗∗	0.148∗∗	0.316∗∗	0.408∗∗	—		
Grit w1	0.190∗∗	0.145∗∗	0.122∗∗	0.219∗∗	0.133∗∗	0.113∗∗	—	
Grit w2	0.138∗∗	0.179∗∗	0.090∗∗	0.148∗∗	0.209∗∗	0.142∗∗	0.440∗∗	—
Grit w3	0.116∗∗	0.124∗∗	0.168∗∗	0.104∗∗	0.117∗∗	0.181∗∗	0.409∗∗	0.467∗∗

^**^p < 0.01, ^*^p < 0.05, w1 = wave 1, w2 = wave 2, w3 = wave 3.

### 3.3 Latent growth model analysis

The latent growth model (LGM) analysis was conducted to examine the initial levels (intercepts) and rates of change (slopes) for exercise time, creative personality, and grit over the three measurement periods. The parameter estimates for the latent growth models of exercise time, creative personality, and grit are summarized in Table 3, while the model fit indices for these models, along with the mediation model, are presented in Table 4. The model fit indices confirmed that all models demonstrated acceptable fit.

**Table 3:** Parameter estimates for latent growth model.

Variables		Mean	*SE*	Variance	*SE*	Covariance	*SE*
Exercise time	intercept	3.072∗∗∗	0.027	1.028∗∗∗	0.079	−0.224 ∗∗∗	0.046
	slope	−0.355∗∗∗	0.016	0.132∗∗∗	0.037		
Creative personality	intercept	1.384∗∗∗	0.059	4.643∗∗∗	0.35	−0.830 ∗∗∗	0.202
	slope	−0.175∗∗∗	0.035	0.702∗∗∗	0.169		
Grit	intercept	2.664∗∗∗	0.008	0.081∗∗∗	0.006	−0.003	0.004
	slope	−0.045∗∗∗	0.005	0.003	0.003		

^***^p < 0.001.

**Table 4:** Model fit indices.

Variables	*χ* ^2^	*df*	*p*	CFI	TLI	RMSEA	SRMR
Exercise time	26.598	1	0	0.97	0.909	0.099	0.0018
Creative personality	9.891	1	0.002	0.991	0.974	0.059	0.0006
Grit	2.849	1	0.091	0.999	0.996	0.027	0.0002
Mediation model	130.408	24	0	0.972	0.958	0.041	-

### 3.4 Exercise time

The analysis revealed that the initial mean level (intercept) of exercise time was 3.072 (*p* < 0.001), with a significant negative rate of change (slope) of −0.355 (*p* < 0.001). The variance of the intercept was 1.028 (*p* < 0.001), indicating significant individual differences in both baseline levels and rates of change. The covariance between the intercept and slope was −0.224 (*C. R.* = − 4.890, *p* < 0.001), with a correlation of −0.607. This significant negative association suggests that students with higher initial levels of exercise time experienced a sharper rate of decline over time.

#### 3.4.1 Creative personality

For creative personality, the initial mean level was 1.384 (*p* < 0.001), and the rate of change was also negative, with a slope of −0.175. The variance of the intercept was 4.643 (*p* < 0.001), and the variance of the slope was 0.702 (*p* < 0.001), showing substantial individual variation in trajectories. The covariance between the intercept and slope was −0.830 (*C. R.* = − 4.116, *p* < 0.001), with a correlation of −0.460. This indicates that individuals starting with higher creative personality scores underwent a significantly steeper decline across the waves.

#### 3.4.2 Grit

The initial mean level of grit was 2.664 (*p* < 0.001), with a slight but significant negative rate of change of −0.045 (*p* < 0.001). The variance for the intercept was 0.081 (*p* < 0.001), reflecting individual differences in baseline grit. Crucially, the variance of the slope was 0.003, which was not statistically significant (*C. R.* = 1.077, *p* = 0.282), indicating that the rate of decline in grit was relatively homogeneous across the sample. Furthermore, the covariance between the intercept and slope was −0.003 and failed to reach statistical significance (*C. R.* = − 0.746, *p* = 0.455, *r* = −0.165). This demonstrates that the baseline level of grit did not systematically influence its subsequent rate of change, diverging from the ‘ceiling effect’ observed in the other constructs.

### 3.5 Mediation model analysis

This study examined the structural longitudinal mediation mechanisms among exercise time, creative personality, and grit using a parallel-process latent growth mediation framework. The analysis rigorously differentiated between the initial baseline values (intercepts) and the longitudinal rates of change (slopes) for each latent construct. Detailed direct path coefficients are structured in Table 5, and the statistical evaluation of indirect and total effects verified via 5,000 bootstrapping iterations is presented in Table 6.

**Table 5:** Path coefficients for latent growth model.

Parameter	Path	*B*	*p*	*SE*
Intercept	Exercise Time→Creative Personality	0.797	<0.001	0.068
	Creative Personality → Grit	0.042	<0.001	0.004
	Exercise Time → Grit	0.081	<0.001	0.01
Slope	Exercise Time → Creative Personality	1.013	<0.001	0.187
	Creative Personality → Grit	0.065	<0.001	0.019
	Exercise Time → Grit	0.045	0.141	0.031

**Table 6:** Indirect effects and total effect.

Path Exercise Time → Creative Personality → Grit		*B*	*p*	*SE*
Intercept	Indirect effect	0.034	0.001	0.005
	Total effect	0.114	0.001	0.01
Slope	Indirect effect	0.066	0.001	0.063
	Total effect	0.111	0.001	0.022

#### 3.5.1 Direct effects

**Figure 1:**
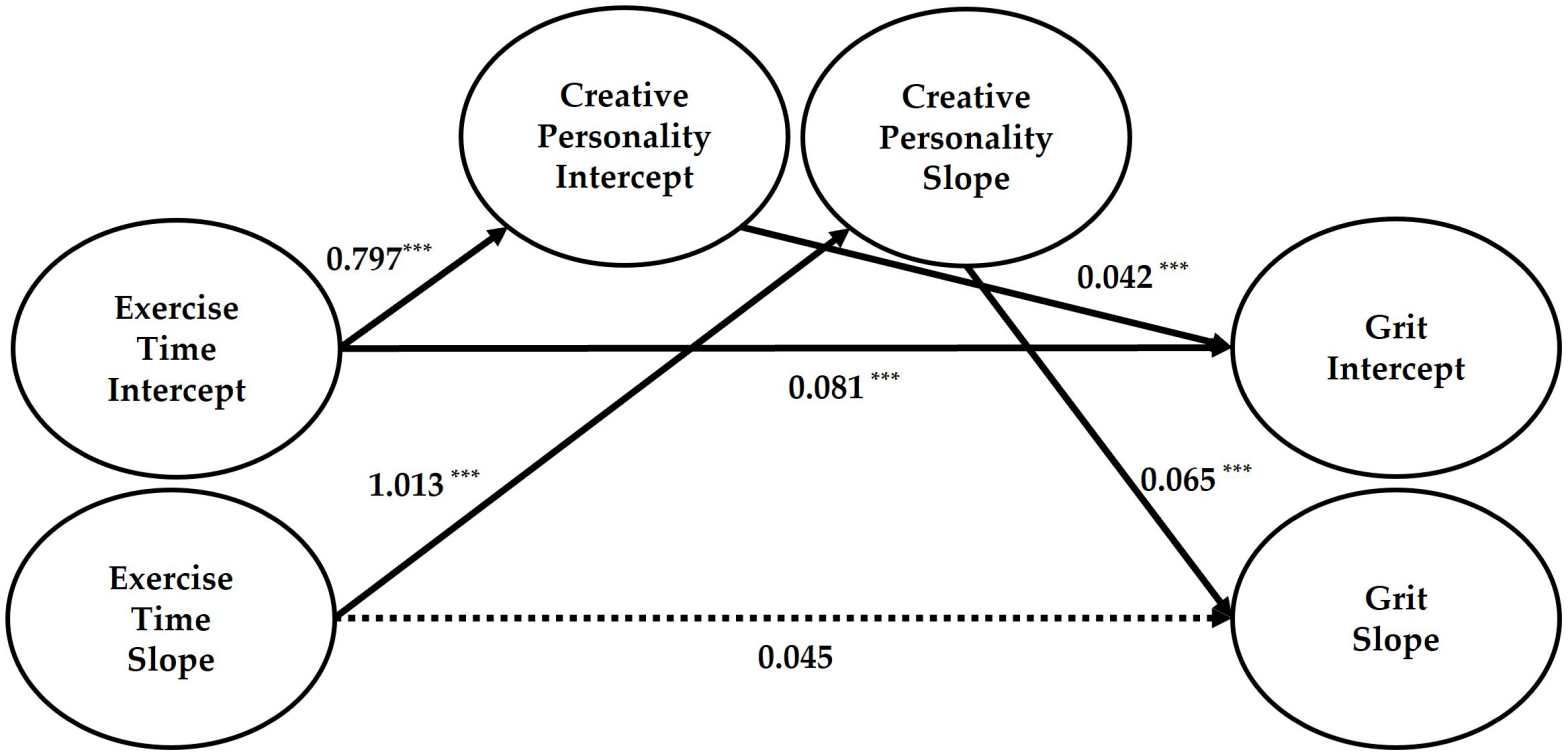
Structural growth mediation model demonstrating the longitudinal pathways among the trajectories of exercise time, creative personality, and grit (*N* = 2,590). Unstandardized coefficients (*B*) are presented. Solid lines indicate statistically significant pathways, whereas the dashed line represents a non-significant direct pathway. ^***^p < 0.001.

The structural path analysis revealed distinct patterns of direct associations across the baseline and growth trajectories. For the intercept pathway, higher initial exercise time significantly and positively predicted higher initial creative personality (*B* = 0.797, *p* < 0.001) and initial grit (*B* = 0.081, *p* < 0.001). Concurrently, higher initial creative personality significantly predicted higher baseline grit (*B* = 0.042, *p* < 0.001). For the slope pathway, a compelling longitudinal dynamic emerged: the rate of change in exercise time significantly and positively predicted the rate of change in creative personality (*B* = 1.013, *p* < 0.001), indicating that a steeper decline in exercise time systematically accelerated the decline in creative personality over the three-year period. Furthermore, the slope of creative personality exerted a strong positive direct effect on the slope of grit (*B* = 0.065, *p* < 0.001). Crucially, however, the direct pathway from the slope of exercise time to the slope of grit failed to reach statistical significance (*B* = 0.045, *p* = 0.260). This demonstrates that while baseline physical activity directly influences initial grit, changes in exercise time over time do not directly drive the trajectory of grit, as visually illustrated by the dashed line in Figure 1.

#### 3.5.2 Indirect effects and total effects

The bootstrapping mediation analysis (Table 6) demonstrated that creative personality serves as a pivotal longitudinal mechanism linking exercise time to grit, yielding structurally different mediation types over time. For the baseline status (the intercept model), the indirect effect of exercise time intercept on grit intercept through creative personality intercept was statistically significant (*B* = 0.034, *p* = 0.001). Since both the direct and indirect pathways remained highly significant, a partial mediation effect was firmly established at the baseline level. Conversely, a structurally distinct full mediation effect (complete mediation) was demonstrated regarding the longitudinal change trajectories (the slope model). The indirect effect of exercise time slope on grit slope via creative personality slope was exceptionally robust and significant (*B* = 0.066, *p* = 0.001). Given that the direct link between the two change slopes was entirely non-significant while this indirect pathway was highly meaningful, the over-time influence of exercise time on grit is completely accounted for by the systematic changes in creative personality. Finally, the total effects of exercise time on grit—which encapsulate the cumulative impact of both direct and indirect structural pathways—were statistically significant across both dimensions. The total effect for the intercept was *B* = 0.114 (*p* = 0.001), and the total effect for the slope was *B* = 0.111 (*p* = 0.001). These comprehensive findings empirically validate that physical activity robustly sustains and enhances grit over time by systematically reinforcing and driving the developmental trajectory of creative personality during the middle school years.

## 4 Discussion

The purpose of this study was to investigate the longitudinal relationships among exercise time, creative personality, and grit using a panel of middle school students. Specifically, latent growth modeling (LGM) was employed to analyze how trajectories of exercise time were associated with grit, both directly and indirectly through creative personality as a mediating variable. The findings provide insights into the developmental trajectories and interconnected mechanisms of these variables during adolescence, revealing trends, longitudinal associations, mediating effects, and practical implications.

### 4.1 Trends in exercise time, creative personality, and grit

The results uncovered distinct trends across the three variables during the transition from early to middle adolescence. Exercise time showed a steady decline over time, consistent with prior research indicating that physical activity levels typically decrease during adolescence due to growing academic responsibilities, reduced access to structured physical activities, and shifting social priorities (10, 15). This decline is particularly concerning, as regular physical activity during adolescence is associated not only with physical health but also with cognitive and emotional well-being. Interventions aimed at supporting physical activity through creative and engaging programs are therefore essential for this age group.

Creative personality similarly exhibited a decreasing trajectory, with notable reductions in both initial levels and growth rates over the study period. This trend may reflect diminishing opportunities for creative exploration as adolescents encounter rigid academic environments that prioritize standardized performance over innovative thinking (19, 45). Additionally, longitudinal research suggests that the transition to higher grade levels often involves increased academic demands and structured curricula, which may constrain the development of adolescents’ creative personality potential (Baer, 1993) (19). This highlights the importance of fostering supportive educational environments that balance creative development alongside academic achievement.

In contrast, grit showed a relatively stable trend, with only a slight decline over time. This stability supports the conceptualization of grit as a relatively enduring trait but one that can still be influenced by environmental factors (1, 41). While the slight decline observed in this study suggests that grit may wane in response to external stressors or reduced opportunities for perseverance-driven tasks, its overall stability underscores its potential for reinforcement through targeted support. These findings collectively emphasize the need for sustained efforts to mitigate the decline of physical activity and creative personality during adolescence, as these variables have far-reaching associations with grit and overall development.

### 4.2 Direct associations of exercise time with creative personality and grit

The analysis revealed significant direct associations between exercise time and creative personality, both at initial levels and over time. Adolescents who engaged in higher levels of physical activity exhibited a greater creative personality, reflecting the link between physical activity and enhanced cognitive processes such as creative personality and problem-solving (16, 19) (Baer, 1993). Physical activities, particularly in dynamic and unpredictable environments like team sports or recreational games, expose adolescents to challenges that may be associated with adaptability and innovative thinking (26, 27). These findings suggest a positive longitudinal association between exercise and creative personality, highlighting its potential to serve as a protective factor for creative personality during adolescence.

The direct association of exercise time with grit yielded mixed results. While higher initial levels of exercise time were significantly associated with greater grit, changes in exercise time over the study period were not directly linked to grit development. This suggests that early engagement in physical activity is positively related to grit by being linked with resilience, goal-setting behaviors, and self-discipline core components of grit (1, 14, 42). However, the nonsignificant association between changes in exercise time and grit emphasizes the multifactorial nature of grit development, which may also depend on factors such as personality, social environments, and intrinsic motivation (15, 17, 28). These findings highlight the need for a more nuanced exploration of how physical activity contributes to grit beyond its direct associations.

### 4.3 The mediating role of creative personality

A key contribution of this study is the empirical verification of creative personality as a significant longitudinal mediator linking exercise time to grit, demonstrating structurally distinct mechanisms across baseline status and rates of change. At the baseline level (the intercept model), a partial mediation effect was established. The indirect effect of exercise time intercept on grit intercept via creative personality intercept was statistically significant (*B* = 0.034). This indicates that at the initial stage of early adolescence, physical activity directly supports components of grit, including self-discipline and goal-directed behaviors (1). Simultaneously, physical activity indirectly reinforces grit by fostering a creative and proactive disposition (19).

Conversely, a more compelling full mediation effect (complete mediation) emerged regarding the longitudinal rates of change over the three-year period (the slope model). While the direct path from the exercise time slope to the grit slope was non-significant (*B* = 0.045), the longitudinal indirect pathway mediated through the creative personality slope was exceptionally robust (*B* = 0.066). This over-time complete mediation carries critical developmental insights: the chronological erosion of grit among middle school students is not a direct consequence of reduced physical activity itself. Rather, a decline in physical activity systematically accelerates the deterioration of their creative personality traits first (*B* = 1.013), which sequentially links to the final longitudinal decline of grit.

Theoretically, creative personality inherently relates to persistence and resilience, particularly during the iterative processes of maintaining and refining novel ideas (19, 43). Engaging in physical activities exposes adolescents to dynamic, unpredictable environments such as team sports or strategic recreational games that continuously stimulate these creative cognitive processes while demanding psychological adaptability and grit (15, 27). Furthermore, creative engagement is closely intertwined with intrinsic motivation, allowing adolescents to explore personal strengths that are vital for sustaining long-term, goal-oriented perseverance (43, 46).

Regarding the methodological framework, our structural inferences are grounded strictly on the unstandardized path coefficients (*B*) and bias-corrected bootstrapping confidence intervals verified through 5,000 iterations (35). In latent growth modeling, when the latent slope variance of a target construct is extremely minute as observed in the grit slope variance of the present study (0.003) standardized path coefficients can become mathematically unstable and artificially inflated. To ensure maximum statistical reliability and avoid psychometric artifacts, this study relied entirely on unstandardized estimates for hypothesis testing. Given that the 95% confidence intervals for the indirect trajectories do not include zero across both baseline and slope dimensions, the longitudinal protective role of creative personality as an intermediate psychological asset is empirically validated as highly reliable.

### 4.4 Implications for practice and policy

The findings of this study carry implications for educational practices and public policy. First, the potential role of creative personality as a mediator emphasizes the need for integrated interventions that combine physical activity with creative personality-enhancing opportunities. Schools and community programs should prioritize activities that buffer the decline of both physical and cognitive development, such as team sports, strategic games, and free play. These activities not only relate to physical health but may also be linked with creative personality and perseverance, contributing to adolescents’ overall development (15, 26, 27).

Second, the observed declines in exercise time and creative personality during adolescence highlight the urgency of sustained efforts to buffer these negative trends. Policymakers should advocate for consistent physical activity throughout middle school by implementing mandatory physical education classes, supporting extracurricular sports programs, and integrating creative tasks into curricula. Such initiatives can address the dual goals of maintaining physical health and mitigating the loss of non-cognitive skills like creative personality and grit (10, 12).

Finally, regarding the interpretation of the growth trajectories, it is necessary to contextualize the statistical attributes of our longitudinal framework. Although the structural path increments appear modest in absolute statistical size, their practical importance must not be overlooked. In a large, nationally representative population (*N* = 2,590), even small effect sizes can translate into substantial structural shifts when accumulated across an entire developmental cohort over time. While the direct longitudinal association between changes in exercise time and grit was non-significant, the indirect pathway was entirely supported through the complete mediation of creative personality. This empirical distinction shifts our practical focus: instead of expecting physical activity to mechanically or directly promote long-term grit, interventions should target creative personality as the critical intermediate outcome. Recognizing this nuanced connection (19, 27), design frameworks that combine physical engagement with creative tasks can yield meaningful cumulative protection against the macro-level decline of adolescent perseverance.

### 4.5 Limitations and future directions

Despite its contributions, this study has several limitations. First, the reliance on self-reported measures, particularly the single-item indicator for exercise time, suggests caution in interpreting the full multi-dimensional variance of the constructs. Specifically, this single-item metric reflects only overall duration, failing to adequately capture the specific frequency, intensity, or type of physical activity. Furthermore, the internal consistency reliability for the grit scale was moderate, which may introduce measurement constraints in tracking longitudinal change. Although longitudinal measurement invariance was conceptually and operationally secured through the standardized panel design of the KCYPS 2018, future research should employ objective measures such as wearable devices to track physical activity and task-based assessments to evaluate creative personality to further enhance the validity and robustness of the findings (28, 42).

Additionally, the structural model did not control for potential socio-demographic covariates, such as socioeconomic status or academic achievement, which could influence the observed developmental relationships. Moreover, because negative trajectories were observed across all primary constructs during the middle school years, these longitudinal relationships should be interpreted as protective effects where physical activity mitigates the rate of developmental decline rather than the absolute promotion of these traits. Finally, the observational nature of the panel data limits the ability to draw definitive causal inferences, warranting longer-term studies to explore how these relationships evolve across subsequent developmental stages.

The nonsignificant slope association of exercise time with grit also warrants further investigation. Future research could examine additional mediating or moderating factors, such as self-efficacy, peer support, or academic experiences, to better understand the mechanisms linking physical activity and grit. Exploring these factors could provide a more comprehensive framework for supporting both cognitive and non-cognitive skill development (17). Lastly, while this study primarily focused on the pathway from physical activity to grit, the relationship between these constructs may be bidirectional. Recent evidence suggests that higher levels of grit can also serve as a precursor that fosters sustained engagement in physical exercise (44). Future research investigating such reciprocal pathways where exercise and grit function as mutually reinforcing assets would offer a more integrated understanding of adolescent development.

## 5 Conclusion

This study provides robust longitudinal evidence for the structural relationships among exercise time, creative personality, and grit in South Korean middle school students. The findings demonstrate that while physical activity is a critical baseline predictor of grit, its over-time protective influence on maintaining adolescents’ perseverance is completely mediated by the developmental trajectory of creative personality traits. This highlights the practical importance of integrating structured physical education and creative asset-enhancing opportunities within school curricula to systematically mitigate the concurrent developmental decline of adolescents’ non-cognitive assets. By addressing the observed reductions in exercise time and creative personality, educators and policymakers can design targeted interventions aimed at supporting the long-term resilience of youth. Future research should continue to explore these reciprocal relationships over extended periods to identify additional multidimensional pathways for supporting grit through holistic, physically engaging experiences.

## Data Availability

Publicly available datasets were analyzed in this study. This data can be found at: https://www.nypi.re.kr/archive.

## References

[1] Duckworth AL Peterson C Matthews MD Kelly DR. Grit: perseverance and passion for long-term goals. J Pers Soc Psychol. (2007) 92:1087–101. doi: 10.1037/0022-3514.92.6.1087 17547490

[2] Duckworth AL Quinn PD. Development and validation of the short grit scale (grit-S). J Pers Assess. (2009) 91:166–74. doi: 10.1080/00223890802634290, 19205937

[3] Eskreis-Winkler L Shulman EP Beal SA Duckworth AL. The grit effect: predicting retention in the military, the workplace, school and marriage. Front Psychol. (2014) 5:36. doi: 10.3389/fpsyg.2014.00036, 24550863 PMC3910317

[4] Strayhorn TL. What role does grit play in the academic success of black male collegians at predominantly white institutions? J Afr Am Stud. (2014) 18:1–10. doi: 10.1007/s12111-012-9243-0

[5] Von Culin KR Tsukayama E Duckworth AL. Unpacking grit: motivational correlates of perseverance and passion for long-term goals. J Posit Psychol. (2014) 9:306–12. doi: 10.1080/17439760.2014.898320, 31404261 PMC6688745

[6] Credé M Tynan MC Harms PD. Much ado about grit: a meta-analytic synthesis of the grit literature. J Pers Soc Psychol. (2017) 113:492–511. doi: 10.1037/pspp0000102, 27845531

[7] Duckworth AL Gross JJ. Self-control and grit: related but separable determinants of success. Curr Dir Psychol Sci. (2014) 23:319–25. doi: 10.1177/0963721414541462, 26855479 PMC4737958

[8] Fredricks JA Eccles JS. Is extracurricular participation associated with beneficial outcomes? Concurrent and longitudinal relations. Dev Psychol. (2006) 42:698–713. doi: 10.1037/0012-1649.42.4.698, 16802902

[9] Little TD. Longitudinal Structural Equation Modeling. 2nd ed. New York, NY: Guilford Publications (2024).

[10] Bailey R Hillman C Arent S Petitpas A. Physical activity: an underestimated investment in human capital? J Phys Act Health. (2013) 10:289–308. doi: 10.1123/jpah.10.3.289, 23620387

[11] Biddle SJH Asare M. Physical activity and mental health in children and adolescents: a review of reviews. Br J Sports Med. (2011) 45:886–95. doi: 10.1136/bjsports-2011-090185, 21807669

[12] Weiss MR Wiese-Bjornstal DM. Promoting positive youth development through physical activity. Presidents Counc Phys Fit Sports Res Dig. (2009) 10:1–8. PMC3022443 21253445

[13] Eime RM Young JA Harvey JT Charity MJ Payne WR. A systematic review of the psychological and social benefits of participation in sport for children and adolescents: informing development of a conceptual model of health through sport. Int J Behav Nutr Phys Act. (2013) 10:98. doi: 10.1186/1479-5868-10-98, 23945179 PMC3751802

[14] Holt NL Deal CJ Pankow K. "Positive youth development through sport". In: Tenenbaum G Eklund RC Boiangin N, editors. Handbook of Sport Psychology: Social Perspectives, Cognition, and Applications, 4th Edn. Hoboken, NJ: John Wiley & Sons, Inc. (2020). p. 429–46.

[15] Bailey R. Physical education and sport in schools: a review of benefits and outcomes. J Sch Health. (2006) 76:397–401. doi: 10.1111/j.1746-1561.2006.00132.x, 16978162

[16] Piya-Amornphan N Santiworakul A Cetthakrikul S Srirug P. Physical activity and creativity of children and youths. BMC Pediatr. (2020) 20:118–7. doi: 10.1186/s12887-020-2017-2, 32164640 PMC7068971

[17] Bollimbala A James PS. Impact of chronic physical activity on individuals' creativity. Psychol Res. (2024) 88:684–94. doi: 10.1007/s00426-023-01862-4, 37561201

[18] González Moreno A Molero Jurado MM. Creativity as a positive factor in the adolescence stage: relations with academic performance, stress, and self-esteem. Behav Sci. (2023) 13:997. doi: 10.3390/bs13120997, 38131853 PMC10740570

[19] Runco MA Acar S. Divergent thinking as an indicator of creative potential. Creat Res J. (2012) 24:66–75. doi: 10.1080/10400419.2012.652929

[20] Said-Metwaly S Taylor CL Camarda A Barbot B. Divergent thinking and creative achievement—how strong is the link? An updated meta-analysis. Psychol Aesthet Creat Arts. (2022) 18:869–81. doi: 10.1037/aca0000507

[21] Saretzki J Forthmann B Benedek M Andrae R Meinzer N Bartlmä L. A systematic Quantitative Review of divergent Thinking Assessments. Psychol Aesthet Creat Arts. (2024). doi: 10.1037/aca0000691

[22] Karwowski M Barbot B. "Creative self-beliefs: their nature, development, and correlates". In: Karwowski M Kaufman JC, editors. The Creative Self. San Diego, CA: Academic Press (2016). p. 27–40.

[23] Kaufman JC Beghetto RA. Beyond big and little: the four C model of creativity. Rev Gen Psychol. (2009) 13:1–12. doi: 10.1037/a0013688

[24] Büning C Jürgens L Lausberg H. Divergent learning experiences in sports enhance cognitive executive functions and creativity in students. Phys Educ Sport Pedagog. (2021) 26:402–16. doi: 10.1080/17408989.2020.1812056

[25] Vestberg T Reinebo G Maurex L Ingvar M Petrovic P. Core executive functions are associated with success in young elite soccer players. PLoS One. (2017) 12:e0170845. doi: 10.1371/journal.pone.0170845, 28178738 PMC5298906

[26] Russ SW Dillon JA. Changes in children's pretend play over two decades. Creat Res J. (2011) 23:330–8. doi: 10.1080/10400419.2011.621824

[27] Santos SD Memmert D Sampaio J Leite N. The spawns of creative behavior in team sports: a creativity developmental framework. Front Psychol. (2016) 7:1282. doi: 10.3389/fpsyg.2016.01282, 27617000 PMC4999444

[28] He Y Yao X Wang S Caughron J. Linking failure feedback to individual creativity: the moderation role of goal orientation. Creat Res J. (2016) 28:52–9. doi: 10.1080/10400419.2016.1125248

[29] Ballerini V Dominici A Ferracane MF Menchetti F Noirjean S. Stimulating creativity and grit of high school students with creative STEM activities: an RCT with noncompliance. Qual Quant. (2024) 59:605–34. doi: 10.1007/s11135-024-01992-w, 30311153

[30] Hass RW Katz-Buonincontro J Reiter-Palmon R. Disentangling creative mindsets from creative self-efficacy and creative identity: do people hold fixed and growth theories of creativity? Psychol Aesthet Creat Arts. (2016) 10:436–46. doi: 10.1037/aca0000081

[31] Yeh YC Lai GJ Lin CF Lin CW Sun HC. How stress influences creativity in game-based situations: analysis of stress hormones, negative emotions, and working memory. Comput Educ. (2015) 81:143–53. doi: 10.1016/j.compedu.2014.09.011

[32] Maxwell SE Cole DA. Bias in cross-sectional analyses of longitudinal mediation. Psychol Methods. (2007) 12:23–44. doi: 10.1037/1082-989X.12.1.23, 17402810

[33] Craig CL Marshall AL Sjöström M Bauman AE Booth ML Ainsworth BE. International physical activity questionnaire: 12-country reliability and validity. Med Sci Sports Exerc. (2003) 35:1381–95. doi: 10.1249/01.MSS.0000078924.61453.FB, 12900694

[34] Milton K Bull FC Bauman A. Reliability and validity of a single-item physical activity measure. Br J Sports Med. (2011) 45:203–8. doi: 10.1136/bjsm.2009.068395, 20484314

[35] Kline RB. Principles and Practice of Structural Equation Modeling. 4th ed. New York, NY: Guilford Press (2015).

[36] Choe I Pyo JM. A study for developing creativity test based on implicit theories of Korean people. Korean J Pers Soc Psychol. (2014) 28:27–47. doi: 10.21193/kjspp.2014.28.1.002

[37] Gough HG. The Adjective check list Manual. Palo Alto, CA: Consulting Psychologists Press (1983).

[38] Kim HM Hwang MH. Validation of the Korean grit scale for children. J Educ. (2015) 35:63–74. doi: 10.25020/je.2015.35.3.63, 33277505

[39] Bollen KA. Latent curve Models: A Structural Equation Perspective. Hoboken, NJ: Wiley-Interscience (2006).

[40] MacKinnon DP Lockwood CM Williams J. Confidence limits for the indirect effect: distribution of the product and resampling methods. Multivar Behav Res. (2004) 39:99–128. doi: 10.1207/s15327906mbr3901_4, 20157642 PMC2821115

[41] Marentes-Castillo M Castillo I Tomás I Álvarez O. Interest and perseverance are not enough to be physically active: the importance of self-efficacy toward healthy eating and healthy weight to move more in adolescents. Sports. (2024) 12:41. doi: 10.3390/sports12020041, 38393261 PMC10893339

[42] Bae MH Zhang X Lee JS. Exercise, grit, and life satisfaction among Korean adolescents: a latent growth modeling analysis. BMC Public Health. (2024) 24:1392. doi: 10.1186/s12889-024-18899-8, 38783255 PMC11119792

[43] Amabile TM. Creativity in context: Update to the social Psychology of Creativity. Boulder, CO: Westview Press (1996).

[44] Hu C Zhang W Huang W Jin C. How grit enhances physical exercise in college students: mediating roles of personal growth initiative and self-efficacy. Front Psychol. (2025) 16:1652984. doi: 10.3389/fpsyg.2025.1652984, 40994858 PMC12455857

[45] Baer J. Creativity and Divergent Thinking: A Task-Specific Approach (1st ed.). Psychology Press. (1993). doi: 10.4324/9781315806785

[46] Yeh Y Lin CS. Achievement goals influence mastery experience via two paths in digital creativity games among elementary school students. Journal of Computer Assisted Learning. (2018). 1–10. doi: 10.1111/jcal.12234, 33277505

